# Elevated Level of Nerve Growth Factor (NGF) in Serum-Derived Exosomes Predicts Poor Survival in Patients with Breast Cancer Undergoing Neoadjuvant Chemotherapy

**DOI:** 10.3390/cancers13215260

**Published:** 2021-10-20

**Authors:** Hae Hyun Jung, Ji-Yeon Kim, Eun Yoon Cho, Jung Min Oh, Jeong Eon Lee, Seok Won Kim, Seok Jin Nam, Yeon Hee Park, Jin Seok Ahn, Young-Hyuck Im

**Affiliations:** 1 Department of Health Sciences and Technology, Samsung Advanced Institute for Health Sciences and Technology, Sungkyunkwan University, Seoul 06351, Korea; junghh0616@gmail.com (H.H.J.); yhparkhmo@skku.edu (Y.H.P.); 2 Samsung Medical Center, Biomedical Research Institute, Sungkyunkwan University School of Medicine, Seoul 06351, Korea; jyeon25.kim@samsung.com (J.-Y.K.); iks1330@gmail.com (J.M.O.); 3 Samsung Medical Center, Department of Medicine, Division of Hematology-Oncology, Sungkyunkwan University School of Medicine, 81 Irwon-ro, Gangnam-gu, Seoul 06351, Korea; ajis@skku.edu; 4 Samsung Medical Center, Department of Pathology, Sungkyunkwan University School of Medicine, Seoul 06351, Korea; eunyoon.cho@samsung.com; 5 Samsung Medical Center, Department of Surgery, Sungkyunkwan University School of Medicine, Seoul 06351, Korea; paojlus@hanmail.net (J.E.L.); seokwon1.kim@samsung.com (S.W.K.); seokjin.nam@samsung.com (S.J.N.)

**Keywords:** exosomes, extracellular vesicles, breast cancer, neoadjuvant chemotherapy, prognostic biomarker, nerve growth factor

## Abstract

**Simple Summary:**

Exosomes and cytokines play crucial roles in the process of tumor progression. Recent studies have reported that cytokines can be packaged into exosomes, leading to drug resistance. The aim of this study is to evaluate the potential value of cytokines in both serum and exosomes as prognostic biomarkers of long-term outcomes in patients with breast cancer treated with neoadjuvant chemotherapy. We observed significant differences in expression patterns between serum cytokines and exosomal cytokines. Elevated levels of serum IP-10, serum MMP-1, and exosomal NGF were associated with poor overall survival. In multivariate analysis, exosomal NGF was an independent prognostic factor for overall survival. These findings suggest that exosomal NGF is useful for identifying patients with poor survival outcomes.

**Abstract:**

Neoadjuvant chemotherapy (NAC) is a standard treatment strategy for patients with locally advanced breast cancer (LABC). However, there are no established predictors of chemosensitivity and survival in LABC patients who undergo NAC. Many studies have demonstrated that exosomes and cytokines are important players in intercellular communication between tumors and their environments, and are involved in chemotherapy resistance. Recently, it was reported that cytokines can be packaged into exosomes, but whether exosomal cytokines serve as biomarkers in breast cancer patients is still unclear. In this study, we examined the roles of cytokines in both serum and exosomes as prognostic biomarkers for long-term outcomes in patients with breast cancer who undergo NAC. We isolated exosomes from the blood of 129 patients with early breast cancer who were receiving neoadjuvant chemotherapy between 2008 and 2011 at Samsung Medical Center. The levels of cytokines and growth factors in serum and exosomes were measured with ProcartaPlex immune-related panels. We investigated correlations between clinic-pathologic variables and patient survival, and Cox proportional hazards regression analysis was performed for prognostic evaluation. We detected significant differences in expression patterns between serum cytokines and exosomal cytokines. In both serum and exosomes, many cytokines were positively correlated with age. In univariate analysis, patients with high serum IP-10, serum MMP-1, and exosomal NGF had shorter overall survival. Exosomal NGF showed significantly poorer overall survival in multivariate analysis. These findings suggest that exosomal NGF is useful for identifying patients with poor survival outcomes.

## 1. Introduction

Breast cancer (BC) is the most common cancer in the world and is the leading cause of cancer death among females [1]. Neoadjuvant chemotherapy (NAC) is a standard treatment strategy for patients with locally advanced breast cancer, especially HER2+ BC and triple negative breast cancer (TNBC). NAC can reduce tumor burden of the breast and decrease tumor size, increasing the chances for successful breast conservation and decreasing the need for axillary node dissection in patients who initially appear to require axillary lymph node dissection. These effects may convert inoperable tumors to operable tumors [2,3]. NAC can eradicate systemic micrometastasis before surgery and be used to evaluate in vivo chemosensitivity, providing valuable information on the effect of systemic chemotherapy on tumor biology [4]. It is important to identify patients who are most likely to have long-term benefits from NAC treatment. Pathologic complete remission (pCR) after NAC is a strong predictor for favorable long-term outcomes in TNBC and HER2+ BC [5,6]. However, patients who do not achieve pCR after NAC represent a heterogeneous group of disease with diverse prognoses, and no reliable biomarkers have been identified to accurately predict prognosis. In addition, patients with hormone receptor (HR) + BC tend to achieve relatively low pCR rates to NAC, and it is difficult to distinguish patients with a good prognosis from those with a poor prognosis [7].

Exosomes, nano-sized extracellular vesicles composed of lipid bilayers, are produced by most cells and contain various biological molecules reflective of their cell types of origin [8,9]. They can mediate communication between cells by transferring cargo molecules such as lipids, nucleic acids, proteins, and cytokines between donor and recipient cells, possibly resulting in changes of the recipient cells. Cancer-associated exosomes can transform normal wild-type cells into malignant cells and drug-sensitive cells into drug-resistant cells [10,11,12]. Many studies have shown that exosomes mediate communication between the tumor microenvironment and the immune system, resulting in increased angiogenesis, metastasis, immune escape, and treatment resistance [13,14].

In addition, accumulating evidence has shown that uptake of tumor-derived exosomes induced by chemotherapy in secondary tissues may modulate tumor behavior, especially in invasion and metastasis [15], host immune response [16], cancer stemness [17], and treatment resistance [12,18]. Several reports suggest that circulating exosomes have predictive value as biomarkers for the response to NAC in patients with breast cancer. One study demonstrated that serum exosomes levels increased in BC patients who underwent NAC, and that the increase of exosomes concentration after NAC was associated with resistance to NAC and poor survival outcomes [19]. Another study reported that the blood levels of breast cancer resistance protein (BCRP) was increased in patients with breast cancers that were resistant to NAC [20]. These data indicate that exosomes are an attractive source for the identification of chemo-resistance factors that could determine the long-term outcomes of patients who receive NAC. Thus, exosomes released by tumor or stromal cells in response to NAC could have predictive value in patients who do not achieve complete response with NAC. However, the prognostic value of circulating exosomes in patients with breast cancer who receive NAC is underexamined.

Cytokine and growth factors are small proteins that have specific roles in intercellular communication, but are also expressed in carcinomas, and are widely recognized as crucial factors in chemoresistance and metastasis through various signaling pathways [21,22]. Cytokines release their soluble forms via the classical endoplasmic reticulum/Golgi pathway. Interestingly, recent studies have found that cytokines can be packaged into exosomes and affect physiological and pathological functions of target cells [23,24]. Several studies have reported that cytokines such as IL-6, IL-8, IL-10, TGF-β, and TNF-α are detected in cancer-associated exosomes, leading to cancer progression and drug resistance [21]. However, the role of exosomal cytokines as potential prognostic biomarkers in patients with breast cancer underwent NAC has never been explored.

The aim of this study is to investigate the potential roles of exosomes and cytokines as prognostic biomarkers of long-term outcomes in BC patients treated with NAC. In this study, we determined the expression of cytokine profiles in both serum and exosomes of BC patients after NAC using multiplex assay-based immune-related panels. We detected significant differences in expression patterns between serum cytokines and exosomal cytokines. We found that many cytokines were positively correlated with age in both serum and exosomes. Ser_IP-10, Ser_MMP-1, and Exo_NGF were associated with clinicopathological factors and predicted OS.

## 2. Materials and Methods

### 2.1. Patients

We retrospectively reviewed the medical data of 200 patients with breast cancer who underwent NAC followed by curative surgery at Samsung Medical Center between 1 July 2008 and 31 December 2011. Patients with bilateral BC, ductal carcinoma in situ (DCIS) and distant metastases were excluded from this study. We included 129 patients with blood samples available at the Samsung Medical Center BioBank in this study.

The clinico-pathological characteristics of tumors, including nuclear and histological grades, tumor size, axillary lymph nodal status, Ki-67 expression, estrogen receptor (ER), progesterone receptor (PgR), and HER2 statuses by immunohistochemical (IHC) staining were assessed by at least two experienced pathologists. ER and PgR positivity were defined as Allred scores in the range of 3–8 according to IHC staining with anti-ER (Immunotech, France) and anti-PgR (Novocastra, UK) antibodies, respectively. HER2 status was evaluated using appropriate antibody staining (DAKO, CA, USA) and/or fluorescence in situ hybridization (FISH). HER2 grades of 0 and 1 were defined as negative results, while grade 3 was identified as a positive result. Amplification of HER2 was confirmed by FISH if HER2 was rated as 2+ by IHC. Ki-67 expression according to IHC analyses were evaluated by both independent semi-quantitative and quantitative methods (DAKO). TNBC was defined as a lack of expression of ER, PgR, and HER2. All tumors were staged based on tumor staging criteria of the seventh edition of the American Joint Committee on Cancer (AJCC).

Regimens in NAC in this study were anthracycline plus cyclophosphamide with subsequent taxane (AC–T) (*n* = 57, 44.2%), followed by anthracycline plus cyclophosphamide (AC) (*n* = 25, 19.4%), anthracycline plus taxane (AT, *n* = 14, 10.9%), anthracycline plus cyclophosphamide with subsequent taxane plus trastuzumab (AC–TH) or other HER2-targeted agent combinations (*n* = 26, 20.2%), (*n* = 17, 13.2%), and other combinations (*n* = 7, 5.4%). Adjuvant chemotherapy was administered in 44 patients (34.1%). Among patients who were HR+ at initial diagnosis (*n* = 83, 64.3%), 78 received adjuvant hormone treatment. All patients who were HER2+ at initial clinical diagnosis (*n* = 39, 30.2%) received adjuvant anti-HER2 treatment for the remaining 1 year.

This study was approved on 11th of January 2018 by the ethics committee of Samsung Medical Center, Seoul, Republic of Korea (IRB No: 2017-12-068) and was conducted in accordance with the guidelines outlined in the Declaration of Helsinki. All experiments were performed in accordance with relevant guidelines and regulations.

### 2.2. Exosome Isolation from Serum

Blood samples were collected immediately prior to curative operation after completion of NAC and were incubated to induce clotting in room temperature for 30 min, then centrifuged at 3000 rpm for 20 min. Serum samples (800 μL) were centrifuged at 2000× *g* for 10 min and centrifuged again at 10,000× *g* at 4 °C for 30 min to completely remove cellular debris. Supernatants from serum were filtered through a filter with a 0.2 μm pore size to remove particles with a size of 0.2 μm or more. The filtered supernatant (700 μL) was centrifuged in an Optima MAX-XP ultracentrifuge with an MLA-130 rotor (Beckman Coulter, Jersey City, NJ, USA) at 100,000× *g* at 4 °C for 1 h for exosome isolation. The pellets concentrated from the filtered supernatant were washed with phosphate buffered saline (PBS), ultracentrifuged again, and dissolved in 70 μL of PBS.

### 2.3. Nanoparticle Tracking Analysis (NTA)

The size distribution and concentration of exosomes were measured using NTA. NanoSight NS300 was used for recording particle movement, and the data were evaluated using NTA v3.2 software (Malvern Instruments, Malvern, UK). The samples were then diluted in 1 mL PBS and thoroughly mixed before being put into the laser chamber. The following settings were used for data collection: detection threshold 3, camera level 14, and acquisition time 30 s.

### 2.4. Nanoview Analysis

The isolated exosomes were incubated overnight with ExoView Tetraspanin Chip (ExoView, Boston, MA, USA) arrayed with antibodies against CD63, CD81 and CD9. Mouse IgG1 was used as a negative control. After washing, the chips were treated with ExoView Tetraspanin Labelling ABs (EV-TC-AB-01) including CD9/ALEXA 488, CD81/ALEXA 555, and CD63/ALEXA 647. Finally, the chips were imaged with the ExoView R100 reader (ExoView) using ExoScan v0.998 acquisition software. The data were analyzed using ExoViewer v0.998 with sizing thresholds set from 50 to 200 nm in diameter.

### 2.5. Transmission Electron Microscopy (TEM)

TEM was performed to confirm the presence and sizes of exosomes. Samples were fixed in 2% paraformaldehyde and placed on formvar-carbon-coated copper grids. Fixed samples were allowed to absorb for 20 min at room temperature and grids were washed with PBS. The samples were fixed again with 2.5% glutaraldehyde for 5 min, washed 10 times with distilled water, and then negatively stained with 1% uranyl acetate for 1 min. Once dry, the images of exosomes were observed using a Hitachi 7700 transmission electron microscope operated at 80 kV.

### 2.6. Western Blot

Proteins were extracted using 10✕ RIPA buffer (Cell Signaling Technology, Danvers, MA, USA). Protein concentration was analyzed by the Micro BCA Protein Assay Reagent Kit (Thermo Scientific, Waltham, MA, USA). Then, 10 µg of proteins per sample were separated on 4–12% Bis-Tris gel (Invitrogen, Waltham, MA, USA) and transferred onto a PVDF membrane (Merck Millipore, Burlington, MA, USA). Membranes was subsequently blocked on and incubated with primary antibodies against CD63 and CD9 (Santa Cruz Biotechnology, Santa Cruz, CA, USA) at 4 °C overnight. After washing three times, membrane was treated with HRP-conjugated antibodies for 1 h at room temperature. Enhanced chemiluminescence (ECL) reagents (Invitrogen) were applied for the detection of proteins according to the manufacturer’s guidelines.

### 2.7. Multiplex Immunoassay

Exosomes and serum biomarker levels were analyzed using Bio-Plex200 (Bio-Rad Laboratories, Hercules, CA, USA) multiplex magnetic bead-based antibody detection kits following the manufacturer’s instructions. The Immune Monitoring 65-Plex Human ProcartaPlex™ Panel and Immuno-Oncology Checkpoint 14-Plex Human ProcartaPlex™ Panel 1 (Invitrogen) were used for detection of a total of 79 analytes.

The first panel (EPX650-10065-901) included the 65 analytes described in previous our report [25]. The second panel (EPX14A-15803-901) included these analytes: BTLA, CD137 (4-1BB), CD152 (CTLA4), CD27, CD28, CD80, GITR, HVEM, IDO, LAG-3, PD-1, PD-L1, PD-L2, and TIM-3. Full names and average levels of studied markers are presented in Table S1.

### 2.8. cBioPortal Analysis

We used the cBioPortal online tool (http://www.cbioportal.org (accessed on 23 April 2021)) [26] to investigate gene alterations and transcriptional regulations of NGF-related genes in BC patients. The Molecular Taxonomy of Breast Cancer International Consortium (METABRIC, 2509 total samples) cohort [27,28,29] was selected for genomics analyses. We selected genomic profiles according to mutations, putative copy-number alterations, and mRNA expression *z*-scores relative to diploid samples (microarray). Of the 2509 patients included, 1904 mRNA gene expression values were available. OncoPrint and Survival data were downloaded after cBioPortal finished its analysis.

### 2.9. Statistical Analysis

SPSS software 25 (IBM Corp., Armonk, NY, USA) and GraphPad Prism 8 (GraphPad Software, San Diego, CA, USA) were used for statistical analysis. Pearson correlation analysis was used to test the relationships between age and expression levels of biomarkers. Comparisons between multiple groups were determined by the Kruskal–Wallis test for nonparametric data. Categorical variables were compared using the chi-square test. The overall survival (OS) period was calculated from the date of diagnosis to the date of death due to any cause. Kaplan–Meier plots for the different groups were compared with the log-rank test. Univariate and multivariate analyses for OS were performed with a Cox proportional hazards model to obtain the hazard ratio (HR) and 95% confidence interval (CI). Statistical significance was two-tailed and considered significant at a *p*-value < 0.05.

## 3. Results

### 3.1. Patient Characteristics

We included 129 patients in this study. Patient characteristics and clinical outcomes are presented in Table 1. The median age at diagnosis was 43 years (range 23–71 years), and 79.1% (*n* = 102) of the patients were premenopausal. The most common histologic subtype was invasive ductal carcinoma (*n* = 125, 96.9%), and 89.1% (*n* = 115) of patients presented with preoperative clinical stage III disease. Forty-two patients (40.3%) were classified as clinical T2 and 59 patients (45.7%) as clinical T3. Clinically, axillary lymph node involvement was present in 123 patients (95.3%). Sixty-one patients (47.3%) had HR+/HER2- BC, 22 patients (17.1%) had HR+/HER2+ BC, 17 patients (13.2%) had HR-/HER2+ BC, and 29 patients (22.5%) had TNBC.

The overall pCR rate after NAC was 7.8% (10 patients). Twenty-six patients (20.2%) were classified as postoperative pathologic stage I, 42 patients (32.6%) as pathologic stage II, and 51 patients (39.6%) as pathologic stage III. Seventy patients (54.3%) presented with nuclear grade 3 (patients, 96.9%), and 47.3% (*n* = 61) of patients presented with histologic grade 2. Fifty-eight patients (45%) had a Ki-67 greater than 20%, whereas 63 patients (48.9%) had a Ki-67 less than 20%.

Within a median follow-up of 5 years (range 0.8 to 7.5), 47 patients (36.4%) had relapsed, including 42 patients (32.6%) with distant relapses and 18 patients with locoregional relapses. Thirty-one patients (24.0%) died during the follow-up period. The survival outcomes according to patient characteristics are listed in Table 1.

### 3.2. Characterization of Isolated Circulating Exosomes

**Figure 1:**
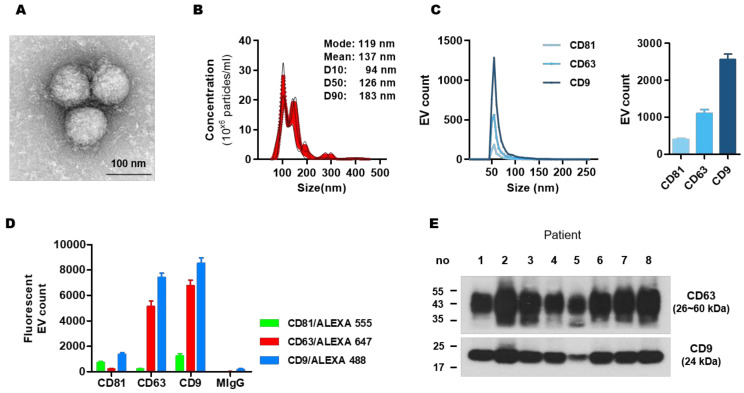
Characterization of exosomes in BC patients. (**A**) Transmission electron microscopic (TEM) observation of exosomes isolated from patients. Scale bar = 100 nm. (**B**) Nanoparticle tracking analysis. The calculated size distribution is depicted as mean (black line) with standard error (red shading). (**C**) ExoView assay was done to measure particle distribution and the number of CD81, CD63, and CD9-positive exosomes. (**D**) Co-expression of exosome markers was measured by probing captured EVs with the indicated secondary fluorescence-labeled antibody. (**E**) Western blot for exosomal protein markers CD63 and CD9 in eight different patients. The uncropped blots of (**E**) were shown in Figure S2.

Recently, we reported that ultracentrifugation is more suitable than the exoEasy and Exoquick methods for the study of exosomal cytokine [25]. Therefore, we used the ultracentrifugation method to isolate exosomes from serum samples of patients. The characterization of exosomes was confirmed. TEM analysis demonstrated round, double-lipid membrane vesicles (Figure 1A). The particle size distribution ranged from 40 to 200 nm in diameter in NTA analysis, with most particles detected in the size range compatible with exosome dimensions (119.4 ± 23.1 nm; Figure 1B). The ExoView assay provided particle size distribution and co-localization of the exosomes with tetraspanins (CD81, CD63, and CD9) (Figure 1C,D). The exosomal markers CD63 and CD9 were present and confirmed by Western blot (Figure 1E). The absence of non-exosomal markers (Calnexin, Histone H3, and β-actin) was confirmed in Figure S1 and the uncropped blots of Figure S1 were shown in Figure S3.

### 3.3. Comparisons of Biomarker Expression Levels in Serum and Exosomes

**Figure 2:**
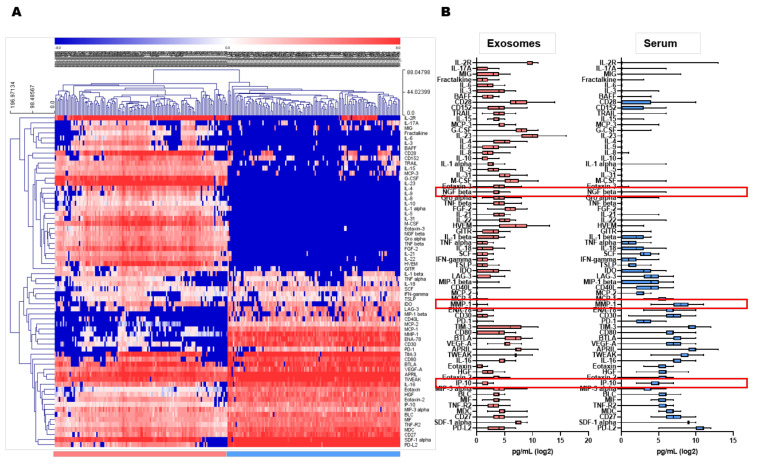
Comparisons of biomarker expression in serum and exosomes by multiplex assay. (**A**) Heat map demonstrating unsupervised hierarchical clustering of samples, generated with Multi-Experiment Viewer (MeV v4.9). (**B**) Box plot demonstrating mean expressions of biomarkers in serum and exosomes.

Next, we performed a multiplex assay using the immune-related panels. Exosomes isolated from 250 µL of patient sera and 25 µL serum were used for multiplex assays. The expression profiles of biomarkers in circulating serum and exosomes are described in Table S1 and Figure 2. The heat map and box plot showed that biomarker profiling differed according to the sample type. Expression levels of analytes were enriched in exosomes compared with serum, although it is necessary to account for the difference in loading volume. 

Twenty-nine analytes (BAFF, CD152, CD28, Eotaxin-3, FGF-2, Fractalkine, G-CSF, GITR, GROα, HVEM, IL-1α, IL-10, IL-15, IL-21, IL-22, IL-23, IL-2R, IL-3, IL-31, IL-4, IL-5, IL-6, IL-8, IL-9, MCP-3, M-CSF, NGF beta, TNF-β, and TRAIL) were mainly enriched in exosomes compared to serum, whereas the concentrations of 27 analytes (APRIL, BLC, CD27, CD30, CD40L, CD80, ENA-78, Eotaxin, Eotaxin-2, HGF, IL-16, IP-10, LAG-3, MCP-1, MCP-2, MDC, MIF, MIP-1β, MMP-1, PD-1, PD-L2, SCF, SDF-1α, TIM-3, TNF-R2, TWEAK, and VEGF-A) were higher in serum than in exosomes. Ten analytes were similarly detected in both exosomes and serum; BTLA, IDO, IFN-γ, IL-1β, IL-17A, IL-18, MIG, MIP-3α, TNF-α, TSLP. The analytes of CD137/4-1BB, GM-CSF, IFN-α, IL-12p70, IL-13, IL-2, IL-20, IL-27, IL-7, I-TAC, LIF, MIP1α, and PD-L1 were not included in this study since the majority (>80% samples) of results were below the limits of assay sensitivity.

### 3.4. Correlations between Biomarker Expression and Age or BC Subtypes

**Figure 3:**
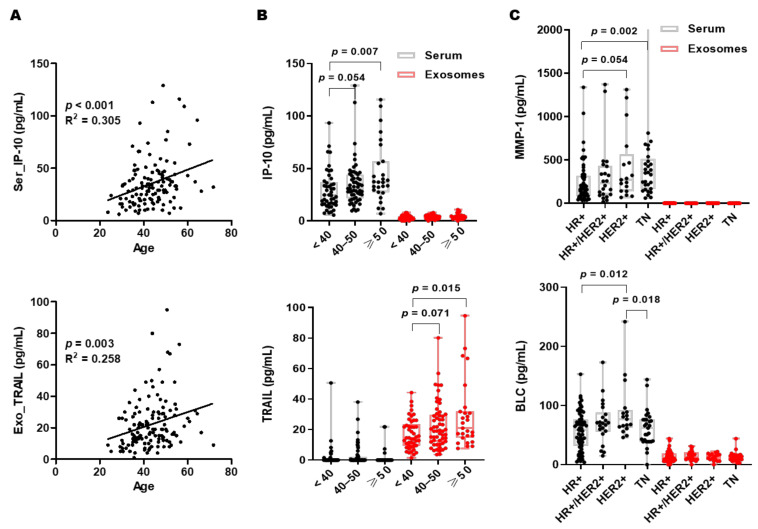
Biomarker expression levels according to age and subtype. (**A**) Correlation plots of age and biomarker level. Boxplots showing the distribution of biomarker levels according to age (**B**) and subtype (**C**).

Pearson’s correlation analysis was performed to determine the relationships between age and the expression levels of biomarkers. As shown in Table 2, the expression levels of 20 biomarkers were correlated with age. We found that serum levels of 10 biomarkers, including HGF, IP-10, Eotaxin, IL-18, BTLA, CD80, SCF, IDO, PD-1, and MCP-1 were positively correlated with age. HGF showed the most significant positive correlation with age (R = 0.375, *p* < 0.001), followed by IP-10 (R = 0.305, *p* < 0.001) and the Kruskal–Wallis test also showed significant results (Figure 3A,B). There were also significant positive correlations between age and 10 biomarkers (TRAIL, Eotaxin-2, TNF-R2, Gro-alpha, Eotaxin-3, BAFF, BLC, MDC, APRIL, and MCP-3) in exosomes. These results suggest that age should be considered when conducting biomarker studies with blood.

We correlated biomarkers with the four molecular subtypes to address the clinical and relevant needs of identifying subgroups. MMP-1 (*p*  =  0.015) and BLC (*p*  =  0.042) in serum showed significant differences between the four groups (Kruskal–Wallis test). Ser_MMP-1 was overexpressed in TNBC subtype vs. HR+ subtype (*p*  =  0.002). Ser_BLC was overexpressed in HER2+ tumors vs. HR+ tumors (*p*  =  0.012) and over-expressed in HER2+ BC vs. TNBC (*p*  =  0.018) (Figure 3C).

**Figure 4:**
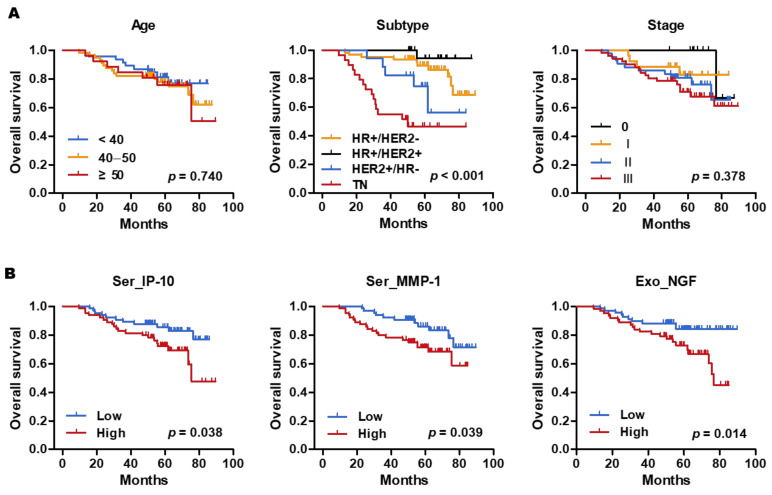
Kaplan–Meier curves for overall survival. (**A**) Kaplan–Meier survival plot for all patients according to age, subtype and stage. (**B**) High levels of Ser_IP-10, Ser_MMP-1, and Exo_NGF were significantly associated with poor OS.

Kaplan-Meier analysis was performed to estimate the correlation of age and subtype with OS. Age was not associated with survival outcomes, but TNBC showed the worst outcome in terms of 5-year OS (48.3%) (Figure 4A).

### 3.5. Prognostic Value of Circulating Serum and Exosomal Biomarkers

To validate the prognostic value of biomarkers, patients were divided into 2 groups with high and low expression based on the median values of each marker. Of all markers, only Ser_IP-10, Ser_MMP-1, and Exo_NGF were significantly associated with poor OS. Kaplan–Meier survival analysis demonstrated that patients with high levels of Ser- IP-10 had poor OS compared to those with low levels of Ser- IP-10 (69% vs. 83%; *p* =  0.038). Likewise, patients with high levels of Ser-MMP-1 presented significantly decreased rates of 5-year OS (69% vs. 83%; *p* = 0.039). In the exosome sample, high NGF expression was significantly associated with poor OS (66% vs. 85%; *p* =  0.014) (Figure 4B).

### 3.6. Relationship between Clinicopathological Factors and Potential Biomarkers

We further investigated the relationships between these selected biomarkers and clinical characteristics (including age, menopausal status, stage, molecular subtype, nuclear grade, histologic grade, and Ki-67). Patients with high levels of Ser-IP-10 were significantly older than those with low levels of Ser_IP-10 (*p* = 0.022), consistent with the results shown in Table 2 and Figure 3. Additionally, overexpression of Ser-IP-10 was associated with unfavorable parameters such as high stage (*p* = 0.049) and high histologic grade (*p* = 0.059). Patients with elevated Ser_MMP-1 expression had more unfavorable parameters, including high nuclear grade (*p* = 0.06), high histologic grade (*p* = 0.005), TNBC subtype (*p* = 0.004), and high Ki-67 score (*p* = 0.005), when compared with patients with low Ser_MMP-1 levels. Unlike Ser_IP-10 and Ser_MMP-1, no correlations were observed between Exo_NGF and clinicopathological factors, of which Exo_NGF appeared to be independent (Table 3).

### 3.7. Exosomal NGF as an Independent Marker of Poor Prognosis in Breast Cancer

We performed Cox proportional hazards regression analysis to discuss the prognostic value of Ser_IP-10, Ser_MMP-1, and Exo_NGF along with key clinicopathological features. Univariate analysis showed that BC subtypes (*p* < 0.001), Ser_IP-10 level (HR = 2.16, 95% CI = 1.02–4.54, *p* =  0.043), Ser_MMP-1 level (HR = 2.13, 95% CI = 1.02–4.47, *p* =  0.044), and Exo_NGF level (HR = 2.5, 95% CI = 1.18–5.31, *p* =  0.017) were significant prognostic factors for OS. Multivariate analysis indicated that BC subtypes (<0.001), stage (*p* =  0.016), and high level of Exo_NGF (HR = 2.41, 95% CI = 1.1–5.27, *p* =  0.027) were independent predictors of poor OS (Table 4). 

### 3.8. Genetic Mutations and Their Associations with Prognosis of NGF-TrkA/p75NTR Axis Genes

We next accessed the cBioPortal tool to evaluate potential correlations between genetic alterations and expressions of the NGF-TrkA/p75NTR axis genes in tumor tissue and prognosis in breast cancer. Using the MAETABRIC database, we explored genetic mutations, putative copy-number alterations, and mRNA expression of NGF-TrkA/p75NTR axis in BC patients.

**Figure 5:**
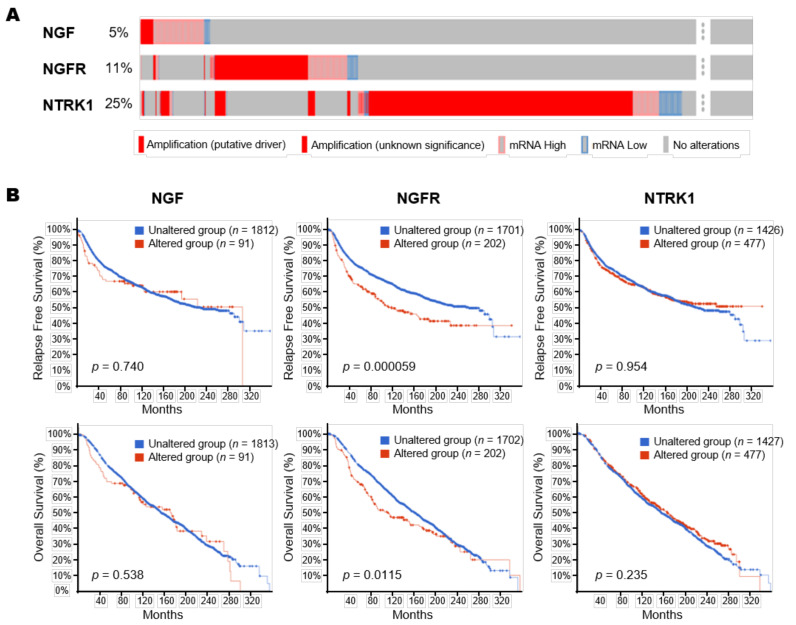
Genetic alterations in NGF-TrkA/p75NTR axis genes and their association with OS and RFS in BC patients (cBioPortal). (**A**) OncoPrint showed genetic alterations of NGF receptor pathway genes from METABRIC samples. (**B**) Kaplan–Meier curves comparing survivals in BC cases with or without genetic alterations in NGF pathway genes.

As shown in Figure 5A, NTRK1 exhibited the highest mutation rate (25%), followed by NGFR (11%) and NGF (5%). DNA copy number amplifications and mRNA up-regulation were the main genetic mutation types. No mutations of the NGF receptor pathway genes were observed in breast cancer. Genetic mutations of NGFR were significantly correlated with poor survival (RFS, *p* < 0.001; OS, *p* = 0.011), whereas mutations of NGF and NTRK1 did not affect survival (Figure 5B). These results suggest that genetic alterations in NGF pathway genes occur at high rates in BC patients and are associated with unfavorable prognosis.

## 4. Discussion

NAC is an increasingly popular treatment strategy in locally advanced breast cancer patients with the goals of achieving tumor downstaging, eradicating distant dissemination, and assessing chemosensitivity in vivo [30]. The most important surrogate marker of long-term outcomes after NAC is pathological complete response (pCR defined as the absence of residual cancer both in the breast and ipsilateral axillary lymph nodes after NAC) [5]. Patients who achieve pCR have significant survival advantages over those who do not, especially patients who are HER2+ BC and TNBC. However, a significant number of patients, including up to 50% of triple-negative (TN) or 30–40% of HER2+ BC and >80% of HR+ BC, do not achieve pCR [31]. Furthermore, although some patients achieved initial pCR, they subsequently relapsed with more advanced disease [32,33]. These observations reflect chemotherapy-induced cancer cell behaviors that contribute to treatment tolerance and tumor cell evolution, and they highlight the need to identify biomarkers to predict and monitor chemotherapy response. Many recent studies have reported that exosomes and cytokines, which are easily available in the peripheral blood, are important players in intercellular communications between tumors and their environments and are involved in chemotherapy resistance.

Fitzgerald and colleagues reported that cytokines can be released in soluble or EV-associated forms in diverse biological systems. Analyses of cytokine distribution revealed that nine cytokines (IL-6, IL-8, IL-13, IL-16, IP-10, MCP-1, MIP-1α, MIP-1β, and MIP-3α) were found more often in the free form. In contrast, eleven cytokines (IL-2, IL-4, IL-12p70, IL-17, IL-21, IL-22, IL-33, IFN-γ, ITAC, TGF-β, and TNF-α) were found in higher levels than the free form in EVs [34]. Similarly, our results show that IL-16, IP-10, MCP-1, and MIP-1α were detected more in serum than in exosomes, and IL-4, IL-21, and IL-22 were higher in exosomes than in serum. Some discrepancies among studies could be due to differences in exosome isolation methods and the presence or absence of chemotherapy.

The aging process is associated with a progressive decline in physiological function and characterized by processes such as cellular senescence and inflammation. Age-related changes in immune response affect the functional properties, composition, and distribution of immune cells, which have important roles in releasing cytokines. Several studies have described a gradual increase in circulating proinflammatory cytokines, concomitant with the decrease in anti-inflammatory mediators associated with increasing age [35]. Recently, Bergen et al. conducted a comprehensive immune biomarker study in BC patients of different ages (35–45, 55–65, ≥70 years). Aging was associated with higher plasma levels of inflammatory cytokines (IL-1α), chemokines (IP-10, IL-8, MCP-1), and immune checkpoint markers (Galectin-9, sCD25, TIM-3) [36]. Here, we obtained similar results. With aging, higher serum levels of 10 biomarkers (HGF, IP-10, Eotaxin, IL-18, TLA, CD80, SCF, IDO, PD-1, and MCP-1) were seen. As in serum, we found that 10 exosomal biomarkers (TRAIL, Eotaxin-2, TNF-R2, Gro-alpha, Eotaxin-3, BAFF, BLC, MDC, APRIL, and MCP-3) also increased with aging. In our patient cohort, there were no significant differences in survival according to age, but age should be considered as a variable in serum biomarker studies.

Our results indicate that elevated serum levels of IP-10, MMP-1 are associated with poor overall survival in univariate analysis. IP-10 (interferon-gamma inducible protein) is a cytokine secreted by several cells in response to IFN-γ. Several studies have demonstrated that overexpression of IP-10 was associated with advanced tumor stages in various types of cancer including breast cancer [37,38]. Recent studies have indicated that BC patients have higher IP-10 than healthy controls and patients with high serum IP-10 had shorter OS [39,40].

Matrix metalloproteinase-1 (MMP1) is a proteolytic enzyme that degrades extracellular matrix. Elevated levels of MMP-1 have been detected in breast cancer and are reported to be associated with breast cancer progression and poor prognosis [41,42]. In addition, some studies found that high MMP1 expression enhances drug resistance [43,44]. Wang et al. demonstrated that MMP-1 expression was associated with breast cancer lymph node metastasis and TNBC. MMP-1 expression was significantly higher in BC patients with axillary lymph node metastasis than without lymph node metastasis and was the highest in TNBC tissues compared to those in HR+ and HER2+ BC tissues [45]. Similarly, our results also indicated that patients with elevated Ser_MMP-1 expression had a high nuclear grade, a high histologic grade, TNBC subtype, and a high Ki-67 score.

Nerve growth factor (NGF) has primarily been investigated in the neurological system, but is also expressed in carcinomas, and has substantial impacts on tumor cell development and metastasis in breast cancer [46]. The binding of ligand NGF with tropomyosin receptor tyrosine kinases A (TrkA) and the TNF-receptor family member p75NTR (NGFR) activates downstream pathways, such as RAS/MAPK and PI3 kinase, resulting in increased cell proliferation and survival of human breast cancer cells [47,48,49,50]. Just as increased blood vessel formation is required for tumor growth, increased nerve density is also one of the components of the tumor microenvironment. Mounting evidence has shown that the nervous system has a role in cancer development and metastasis. Nerve fibers around the tumor release neurotransmitters into surrounding tissues affecting on receptors expressed by tumor cells [51]. Cancer-associated neurogenesis, called tumor innervation, has also been reported in breast cancer and is associated with aggressive clinical characteristics including tumor grade, poor survival outcome [52,53].

The present study demonstrates the presence of exosomal NGF and their association with poor prognosis in BC patients treated with NAC. Although we were unable to compare exosomal NGF levels in the serum with corresponding tumor tissue, we probed public databases to determine the associations of the NGF-TrkA/p75NTR axis with survival outcomes in patients with breast cancer. Our results indicate that NGF-TrkA/p75NTR had alterations, mainly DNA copy number amplifications and mRNA, with alterations correlating with worse survival. To the best of our knowledge, this is the first study to report the prognostic significance of exosomal NGF in breast cancers.

In this study, the patients were mainly premenopausal, reflecting the younger population in the Asia Pacific region compared to Western countries, and characterized by high risk of relapse with advanced stages (clinical stage II 11%, clinical stage III 89%) at the time of diagnosis before NAC. Limitations of our study are its retrospective nature and inclusion of a relatively small number of patients and heterogeneous subtypes of breast cancer. Prospective studies with large numbers of patients are warranted to validate the usefulness of this potential biomarker in breast cancer treated with NAC.

## 5. Conclusions

In summary, we examined the roles of cytokines in both serum and exosomes as prognostic biomarkers for long-term outcomes in patients with breast cancer who were treated with NAC. We observed significant differences in expression patterns between serum cytokines and exosomal cytokines. In both serum and exosomes, many cytokines were positively correlated with age. In univariate analysis, patients with high serum IP-10, serum MMP-1 and exosomal NGF showed poor overall survival compared with low groups. Further, exosomal NGF showed significantly poor overall survival in multivariate analysis. These findings suggest that exosomal NGF might be useful for predicting survival outcomes in patients with breast cancer under neoadjuvant therapy. Further studies to understand the functional mechanisms of exosomal NGF are warranted and will aid in the development of prevention and treatment strategies. 

## Figures and Tables

**Table 1:** Patient characteristics and clinical outcomes.

Characteristics	Total (*n* = 129), *n* (%)	5Y OS (%)	*p*-Value
Age, years			
<40	46 (35.7)	80.4	0.74
40–50	56 (43.5)	73.2	
≥50	27 (21)	74.1	
Menopausal status			
Postmenopausal	23 (17.9)	69.6	0.453
Perimenopausal	3 (2.4)	100	
Premenopausal	102 (79.1)	76.5	
Unknown	1 (0.8)		
Clinical Stage			
II	14 (10.9)	100	0.037
III	115 (89.2)	73.0	
Clinical T stage			
1	2 (1.6)	100	0.669
2	52 (40.4)	82.7	
3	59 (45.8)	69.5	
4	16 (12.5)	75	
Clinical N stage			
0	6 (4.7)	100	0.39
1	18 (14)	72.2	
2	70 (54.3)	78.6	
3	35 (27.2)	68.6	
Molecular subtype			
HR+, HER2−	61 (47.3)	83.6	<0.001
HR+, HER2+	22 (17.1)	95.5	
HR−, HER2+	17 (13.2)	70.6	
TN (HR−, HER2−)	29 (22.5)	48.3	
Pathologic Stage (AJCC 7th)			
0	10 (7.8)	90	0.378
I	26 (20.2)	84.6	
II	42 (32.6)	76.2	
III	51 (39.6)	68.6	
Nuclear grade (NG)			
1	18 (14)	88.9	0.071
2	40 (31.1)	85.0	
3	70 (54.3)	68.6	
Unknown	1 (0.8)		
Histologic grade (HG)			
1	25 (19.4)	96	<0.001
2	61 (47.3)	80.3	
3	31 (24.1)	48.4	
Unknown	12 (9.4)		
Ki-67 score			
≤20%	63 (48.9)	88.9	<0.001
20%<	58 (45)	58.6	
Unknown	8 (6.3)		

Overall survival (OS) was determined by Kaplan–Meier analysis and the log-rank test. *p*-values ≤ 0.05 were considered significant.

**Table 2:** Relationships between biomarker expression and age.

Variable	Pearson Correlation Test		≤40 Years	40 < Age ≤ 50	>50 Years	Kruskal Wallis Test
	R2	*p*-Value	Mean ± SD	Mean ± SD	Mean ± SD	*p*-Value
Age	1	-	34.6 ± 3.8	45 ± 3.1	55.7 ± 5.5	<0.001
Ser_HGF	0.375	<0.001	55.6 ± 39.8	62.2 ± 37.6	117.5 ± 107.4	<0.001
Ser_IP-10	0.305	<0.001	29.2 ± 18.8	35.5 ± 21.6	45.7 ± 29.1	0.014
Ser_Eotaxin	0.253	0.004	39.9 ± 24.2	43.4 ± 24.1	53 ± 20.3	0.008
Ser_IL-18	0.229	0.009	5.2 ± 7.8	6.2 ± 11.4	12.5 ± 15.1	0.017
Ser_BTLA	0.223	0.011	181.2 ± 130.4	192.5 ± 99.9	274 ± 193.4	0.042
Ser_CD80	0.204	0.02	118.8 ± 83.1	131.8 ± 72.6	212 ± 163.4	0.003
Ser_SCF	0.201	0.022	9.1 ± 6.4	11.4 ± 7.5	14.1 ± 6.6	0.006
Ser_IDO	0.198	0.024	8.5 ± 8.9	12.4 ± 12.2	15 ± 11.2	0.032
Ser_PD-1	0.193	0.029	9.2 ± 9.9	11.2 ± 8.5	15.2 ± 15.4	0.039
Ser_MCP-1	0.186	0.035	40.4 ± 24.5	50 ± 23.9	58 ± 33	0.023
Exo_TRAIL	0.258	0.003	17.2 ± 10	23 ± 15.2	29.2 ± 22.4	0.036
Exo_Eotaxin-2	0.246	0.005	9.9 ± 7.7	13.4 ± 9.5	15.4 ± 10.6	0.047
Exo_TNF-R2	0.232	0.008	7.5 ± 5.1	10.3 ± 8.2	12.8 ± 10.5	0.036
Exo_Gro alpha	0.221	0.012	17.4 ± 16.5	24 ± 21.6	34.1 ± 40.5	0.044
Exo_Eotaxin-3	0.213	0.015	18.4 ± 19.9	31.8 ± 32.2	37.9 ± 52.4	0.035
Exo_BAFF	0.211	0.017	4.5 ± 4.2	5.5 ± 4.5	7.3 ± 4.5	0.023
Exo_BLC	0.195	0.027	11.5 ± 7.8	13.9 ± 7.7	16.1 ± 8.6	0.022
Exo_MDC	0.195	0.027	23.8 ± 18	32.1 ± 28.8	47.2 ± 68.7	0.042
Exo_APRIL	0.191	0.030	141.8 ± 85.6	232.7 ± 226.7	289 ± 334.3	0.022
Exo_MCP-3	0.183	0.038	17 ± 6.8	19.9 ± 12.4	21.7 ± 8.5	0.047

**Table 3:** Associations of serum IP-10, serum MMP-1 and exosomal NGF levels with clinical characteristics.

Characteristics	Ser_IP-10			Ser_MMP-1			Exo_NGF beta		
	Low *n* (%)	High *n* (%)	*p*	Low *n* (%)	High *n* (%)	*p*	Low *n* (%)	High *n* (%)	*p*
Age, years			0.022 *			0.118			0.705
<40	29 (45)	17 (27)		20 (31)	26 (41)		26 (39)	20 (32)	
40–50	28 (43)	28 (44)		34 (52)	22 (34)		27 (40)	29 (47)	
≥50	8 (12)	19 (30)		11 (17)	16 (25)		14 (21)	13 (21)	
Menopausal status			0.106			0.122			0.621
Premenopausal	7 (11)	16 (25)		9 (14)	14 (22)		10 (15)	13 (21)	
Perimenopausal	2 (3)	1 (2)		3 (5)	0 (0)		2 (3)	1 (2)	
Postmenopausal	55 (86)	47 (73)		53 (82)	49 (78)		54 (82)	48 (77)	
Clinical Stage			0.68			0.271			0.095
II	8 (12)	6 (10)		9 (14)	5 (8)		10 (15)	4 (6)	
III	59 (88)	56 (90)		56 (86)	59 (92)		55 (85)	60 (94)	
Molecular subtype			0.681			0.004 *			0.339
HR+, HER2−	33 (51)	28 (44)		40 (62)	21 (33)		33 (49)	28 (45)	
HR+, HER2+	12 (18)	10 (16)		11 (17)	11 (17)		14 (21)	8 (13)	
HR−, HER2+	8 (12)	9 (14)		6 (9)	11 (17)		6 (9)	11 (18)	
TN (HR−, HER2−)	12 (18)	17 (27)		8 (12)	21 (33)		14 (21)	15 (24)	
Stage (AJCC 7th)			0.049 *			0.601			0.506
0	8 (12)	2 (3)		3 (5)	7 (11)		4 (6)	6 (10)	
I	8 (12)	18 (28)		13 (20)	13 (20)		14 (21)	12 (19)	
II	23 (35)	19 (30)		22 (34)	20 (31)		19 (28)	23 (37)	
III	26 (40)	25 (39)		27 (42)	24 (38)		30 (45)	21 (34)	
Nuclear grade			0.664			0.06			0.783
1	10 (15)	8 (13)		12 (18)	6 (10)		9 (14)	9 (15)	
2	22 (34)	18 (29)		24 (37)	16 (25)		19 (29)	21 (34)	
3	33 (51)	37 (59)		29 (45)	41 (65)		38 (58)	32 (52)	
Histologic grade			0.059			0.005 *			0.874
1	16 (29)	9 (15)		20 (33)	5 (9)		13 (21)	12 (21)	
2	30 (54)	31 (51)		29 (48)	32 (57)		33 (54)	28 (50)	
3	10 (18)	21 (34)		12 (20)	19 (34)		15 (25)	16 (29)	
Ki-67			0.176			0.005 *			0.541
≤20%	36 (58)	27 (46)		39 (65)	24 (39)		35 (55)	28 (49)	
20%<	26 (42)	32 (54)		21 (35)	37 (61)		29 (45)	29 (51)	

*p*-value < 0.05 is significant. Asterisks denote significance.

**Table 4:** Cox regression analysis for overall survival (OS).

Variable	Univariate Analysis		Multivariate Analysis	
	Hazard Ratio (95% CI)	*p*-Value	Hazard Ratio (95% CI)	*p*-Value
Age, years		0.741		
<40	ref			
40-50	1.34 (0.58−3.06)			
≥50	1.4 (0.52−3.75)			
Molecular subtype		<0.001		<0.001
HR+, HER2-	ref		ref	
HR+, HER2+	0.28 (0.04−2.2)		0.46 (0.06−3.62)	
HR−, HER2+	2.2 (0.75−6.48)		3.2 (1.02−10.06)	
TN (HR−, HER2−)	6.03 (2.64−13.81)		9.36 (3.85−22.78)	
Pathologic Stage		0.418		0.016
0	ref		ref	
I	2.08 (0.23−18.74)		2.04 (0.22−18.57)	
II	3.21 (0.41−25.19)		3.41 (0.42−27.89)	
III	3.98 (0.53−30.04)		7.97 (1.02−62.58)	
Ser_IP-10		0.043		
Low	ref			
High	2.16 (1.02−4.54)			
Ser_MMP-1		0.044		
Low	ref			
High	2.13 (1.02−4.47)			
Exo_NGF		0.017		0.027
Low	ref		ref	
High	2.5 (1.18−5.31)		2.41 (1.1−5.27)	

Abbreviations: HR, hazard ratio; CI, confidence interval.

## Data Availability

The data presented in this study are available on request from the corresponding author.

## Supplementary Materials

The following are available online at https://www.mdpi.com/article/10.3390/cancers13215260/s1, Figure S1: Characterization of exosome preparation by Western blot, Figure S2: Uncut blots for Figure 1E, Figure S3: Uncut blots for Figure S1, Table S1: Full name for each target in the ProcartaPlex immune-related Panel.
