# Supplementary material for: Elevated Level of Nerve Growth Factor (NGF) in Serum-Derived Exosomes Predicts Poor Survival in Patients with Breast Cancer Undergoing Neoadjuvant Chemotherapy
Source: Cancers (Basel). 2021 Oct 20;13(21):5260. doi: 10.3390/cancers13215260 (PMC8582365; doi:10.3390/cancers13215260)
Supplement: Supplementary file 1 [file cancers-13-05260-s001.zip › cancers-1349897-supplementary.pdf]

Supplementary Materials

Elevated Level of Nerve Growth Factor (NGF) in Serum-Derived Exosomes Predicts Poor Survival in Patients with Breast Cancer Undergoing Neoadjuvant Chemotherapy

Hae Hyun Jung, Ji-Yeon Kim, Eun Yoon Cho, Jung Min Oh, Jeong Eon Lee, Seok Won Kim, Seok Jin Nam, Yeon Hee Park, Jin Seok Ahn and Young-Hyuck Im

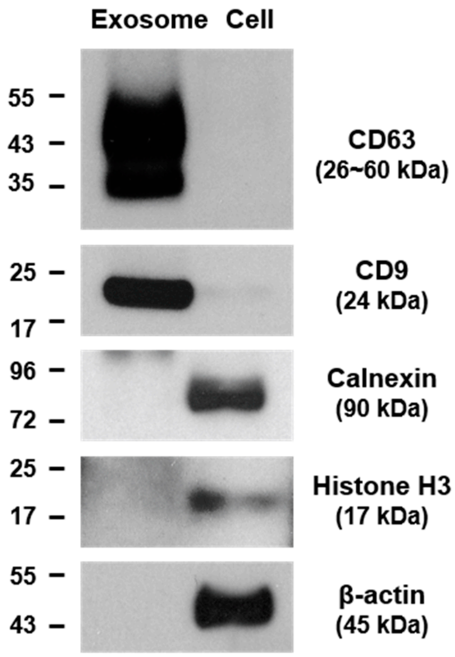

Figure S1. Characterization of exosome preparation by Western blot. Exosomes were isolated as described in Section 2.2. in Materials and Methods. The lysate of MDA-MB-231 cell line was loaded as control. The equal amounts of protein samples (10 μg protein) were subjected to SDS-PAGE, followed by immunoblot with the indicated antibodies. The presence of exosomal markers (CD63 and CD9), and the absence of non-exosomal markers (Calnexin, Histone H3, and β-actin) demonstrates a pure exosome preparation.

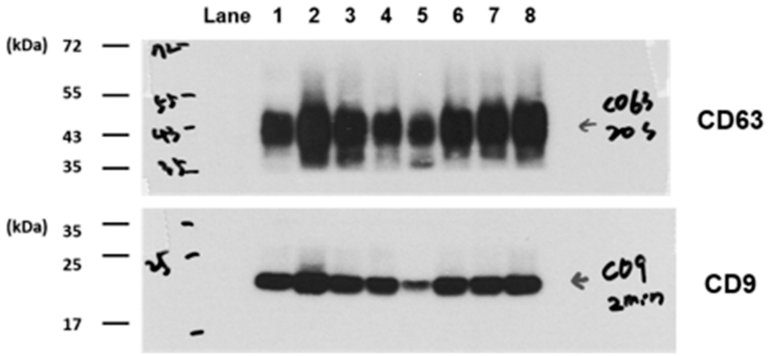

Band area density		
Ab	CD63	CD9
Lane 1	64,373	66,810
Lane 2	112,889	36,567
Lane 3	88,838	29,556
Lane 4	60,366	25,690
Lane 5	58,451	66,799
Lane 6	80,926	66,812
Lane 7	90,119	32,691
Lane 8	99,121	43,392

Figure S2. Uncut blots for Figure 1E.

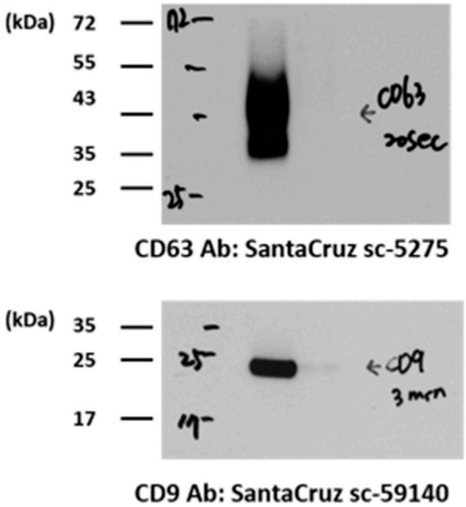

Band area density		
Ab	Lane1	Lane2
CD63	123,985	2,050
CD9	32,558	1,052
Calnexin	88	26,432
Histone H3	1,947	19,944
β-actin	49	32,922

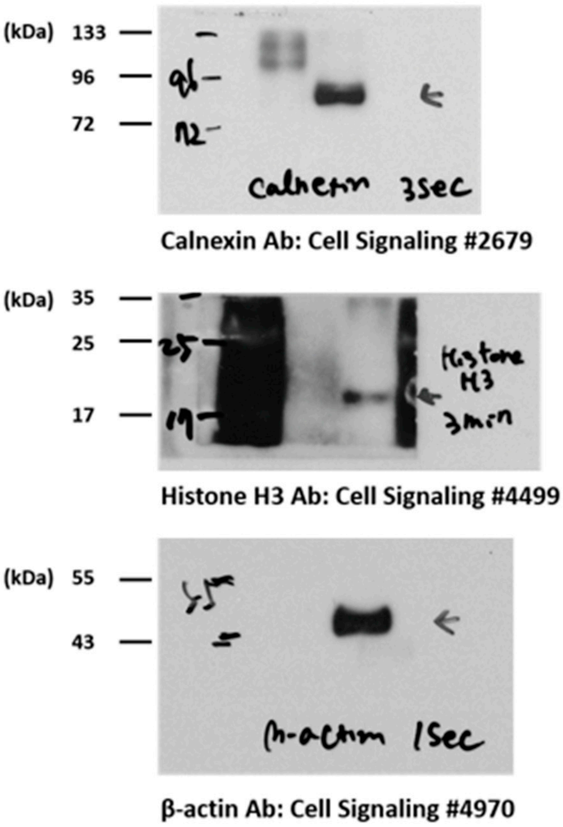

Figure S3. Uncut blots for Figure S1.

Table S1. Full name for each target in the ProcartaPlex immune-related Panel.

Analyte	Gene Name (HGNC)	Protein Name (Uniprot)	Exosomes (Mean ± SD)	Serum (Mean ± SD)
APRIL	TNFSF13	Tumor necrosis factor ligand superfamily member 13	212.1 ± 224.9	933.8 ± 727.4
BAFF	TNFSF13B	Tumor necrosis factor ligand superfamily member 13B	5.5 ± 4.5	0.6 ± 2.1
BLC	CXCL13	C-X-C motif chemokine 13	13.5 ± 8	65.1 ± 36.6
BTLA	BTLA	B- and T-lymphocyte attenuator	151.4 ± 155	205.5 ± 138.4
CD137/4-1BB	TNFRSF9	Tumor necrosis factor receptor superfamily member 9	1.1 ± 3.7	2.7 ± 6.5
CD152	CTLA4	Cytotoxic T-lymphocyte protein 4	30.6 ± 46.9	4.9 ± 8.3
CD27	CD27	CD27 antigen	27.8 ± 47.9	152.5 ± 130.8
CD28	CD28	T-cell-specific surface glycoprotein CD28	543.2 ± 1349.4	21 ± 95.8
CD30	TNFRSF8	Tumor necrosis factor receptor superfamily member 8	1.7 ± 2.2	170.2 ± 137
CD40L	CD40LG	CD40 ligand	1.3 ± 6.3	20.6 ± 22.6
CD80	CD80	T-lymphocyte activation antigen CD80	19.9 ± 20.3	144 ± 106.7
ENA-78	CXCL5	C-X-C motif chemokine 5	0.7 ± 1.4	134.3 ± 112.9
Eotaxin	CCL11	Eotaxin	0.8 ± 0.7	44.2 ± 23.7
Eotaxin-2	CCL24	C-C motif chemokine 24	12.5 ± 9.3	64.9 ± 31.2
Eotaxin-3	CCL26	C-C motif chemokine 26	28.3 ± 34.7	0.1 ± 0.3
FGF-2	FGF2	Fibroblast growth factor 2	100.1 ± 86.4	-
Fractalkine	CX3CL1	Fractalkine, C-X3-C motif chemokine ligand 1	2.5 ± 2.8	0.3 ± 1.2
G-CSF	CSF3	Granulocyte colony-stimulating factor	324.8 ± 381.1	0.4 ± 2.5
GITR	TNFRSF18	Tumor necrosis factor receptor superfamily member 18	8.7 ± 8.4	0.4 ± 1.8
GM-CSF	CSF2	Granulocyte-macrophage colony-stimulating factor	7.2 ± 11.4	1.8 ± 15.1
GRO-alpha	CXCL1	Growth-regulated alpha protein	23.8 ± 25.8	0.3 ± 2.9
HGF	HGF	Hepatocyte growth factor	4.3 ± 4.1	71.4 ± 63.9
HVEM	TNFRSF14	Tumor necrosis factor receptor superfamily member 14	378.1 ± 971	0.1 ± 0.9
IDO	IDO1	Indoleamine 2,3-dioxygenase 1	10.3 ± 10	11.5 ± 11.1
IFN-alpha		Interferon alpha	1.5 ± 1.2	0.1 ± 0.4
IFN-gamma	IFNG	Interferon gamma	2.2 ± 2.1	3.2 ± 3.9
IL-1 alpha	IL1A	Interleukin-1 alpha	7.6 ± 5.1	0.7 ± 5.8
IL-1 beta	IL1B	Interleukin-1 beta	7.8 ± 7.8	4.6 ± 4.9
IL-10	IL10	Interleukin-10	2.9 ± 3	-
IL-12p70		Interleukin 12p70	1.2 ± 0.9	1.4 ± 0.9
IL-13	IL13	Interleukin-13	0.6 ± 0.8	0.1 ± 0.1
IL-15	IL15	Interleukin-15	12.5 ± 5.1	0.5 ± 1.4
IL-16	IL16	Pro-interleukin-16	26 ± 19.2	155.3 ± 86.5
IL-17A	IL17A	Interleukin-17A	3.2 ± 4.3	2.2 ± 6.6
IL-18	IL18	Interleukin-18	4.4 ± 4.9	7.2 ± 11.5
IL-2	IL2	Interleukin-2	2.2 ± 3.3	3.8 ± 11.4
IL-20	IL20	Interleukin-20	2.2 ± 10	3.5 ± 30.2
IL-21	IL21	Interleukin-21	18 ± 13.2	0.7 ± 3.7
IL-22	IL22	Interleukin-22	36.8 ± 24.5	1.1 ± 5.9
IL-23		Interleukin-23	2968.2 ± 6362.5	-
IL-27		Interleukin-27	4 ± 12.2	-
IL-2R		Interleukin-2 receptor	775.4 ± 464.5	175.1 ± 657.4
IL-3	IL3	Interleukin-3	20 ± 25.2	0.5 ± 3.5
IL-31	IL31	Interleukin-31	66 ± 77.2	-
IL-4	IL4	Interleukin-4	44.5 ± 51.7	-
IL-5	IL5	Interleukin-5	15.5 ± 18.8	-
IL-6	IL6	Interleukin-6	6.7 ± 7.9	-
IL-7	IL7	Interleukin-7	0.1 ± 0.2	0.5 ± 0.8
IL-8	CXCL8	Interleukin-8	5.8 ± 7.3	0.1 ± 0.3
IL-9	IL9	Interleukin-9	16.3 ± 22.1	-
IP-10	CXCL10	C-X-C motif chemokine 10	3.7 ± 2.1	35.4 ± 23.1
I-TAC	CXCL11	C-X-C motif chemokine 11	6.9 ± 15	8.3 ± 29.7

LAG-3	LAG3	Lymphocyte activation gene 3 protein	2.9 ± 4.8	22.1 ± 19.5
LIF	LIF	Leukemia inhibitory factor	0.4 ± 0.8	0.1 ± 0.3
MCP-1	CCL2	C-C motif chemokine 2	0.5 ± 1	48.3 ± 26.9
MCP-2	CCL8	C-C motif chemokine 8	0.1 ± 0.2	8.2 ± 4.6
MCP-3	CCL7	C-C motif chemokine 7	19.2 ± 10	3.1 ± 7
M-CSF	CSF1	Macrophage colony-stimulating factor 1	149.6 ± 202.9	0.4 ± 4.5
MDC	CCL22	C-C motif chemokine 22	32.3 ± 38.8	111 ± 51
MIF	MIF	Macrophage migration inhibitory factor	13.9 ± 10.5	45.2 ± 13.5
MIG	CXCL9	C-X-C motif chemokine 9	10.6 ± 13.9	6.2 ± 26.9
MIP-1 alpha	CCL3	C-C motif chemokine 3	0.5 ± 0.9	1.1 ± 2.4
MIP-1 beta	CCL4	C-C motif chemokine 4	0.3 ± 1.4	20.7 ± 25
MIP-3 alpha	CCL20	C-C motif chemokine 20	35 ± 68.3	16 ± 9.7
MMP-1	MMP1	Interstitial collagenase	0.5 ± 0.7	322.7 ± 351.4
NGF beta	NGF	Beta-nerve growth factor	16.8 ± 9.1	0.5 ± 5
PD-1	PDCD1	Programmed cell death protein 1	0.7 ± 1.8	11.3 ± 10.9
PD-L1	CD274	Programmed cell death 1 ligand 1	1 ± 1.1	0.6 ± 0.8
PD-L2	PDCD1LG2	Programmed cell death 1 ligand 2	16.4 ± 15.1	1730.5 ± 719.7
SCF	KITLG	Kit ligand	2.1 ± 2.1	11.2 ± 7.1
SDF-1 alpha	CXCL12	stromal cell-derived factor 1 alpha	160.1 ± 110.5	604.3 ± 157.7
TIM-3	HAVCR2	Hepatitis A virus cellular receptor 2	184.4 ± 270.9	666.1 ± 432.8
TNF alpha	TNF	Tumor necrosis factor	2.1 ± 2.3	2.7 ± 2.5
TNF beta	LTA	Lymphotoxin-alpha	31.1 ± 36	-
TNF-R2	TNFRSF1B	Tumor necrosis factor receptor superfamily member 1B	9.8 ± 8	48.9 ± 20.4
TRAIL	TNFSF10	Tumor necrosis factor ligand superfamily member 10	22.2 ± 16	2.5 ± 7.2
TSLP	TSLP	Thymic stromal lymphopoietin	2.6 ± 2.9	3.2 ± 2.5
TWEAK	TNFSF12	Tumor necrosis factor ligand superfamily member 12	151.9 ± 90.7	433.5 ± 295.1
VEGF-A	VEGFA	Vascular endothelial growth factor A	54.2 ± 40.1	161.3 ± 153.1
